# Individual Differences in Motor Timing and Its Relation to Cognitive and Fine Motor Skills

**DOI:** 10.1371/journal.pone.0069353

**Published:** 2013-07-09

**Authors:** Håvard Lorås, Ann-Katrin Stensdotter, Fredrik Öhberg, Hermundur Sigmundsson

**Affiliations:** 1 Faculty of Health Education and Social Work, Division Physiotherapy, Sør-Trøndelag University College, Trondheim, Norway; 2 Research Group for Learning and Skill Development, Department of Psychology, Norwegian University of Science and Technology, Trondheim, Norway; 3 Department of Biomedical Engineering and Informatics, Radiation Science, Umeå University, Umeå, Sweden; Duke University, United States of America

## Abstract

The present study investigated the relationship between individual differences in timing movements at the level of milliseconds and performance on selected cognitive and fine motor skills. For this purpose, young adult participants (*N* = 100) performed a repetitive movement task paced by an auditory metronome at different rates. Psychometric measures included the digit-span and symbol search subtasks from the Wechsler battery as well as the Raven SPM. Fine motor skills were assessed with the Purdue Pegboard test. Motor timing performance was significantly related (mean *r* = .3) to cognitive measures, and explained both unique and shared variance with information-processing speed of Raven's scores. No significant relations were found between motor timing measures and fine motor skills. These results show that individual differences in cognitive and motor timing performance is to some extent dependent upon shared processing not associated with individual differences in manual dexterity.

## Introduction

In the catalogue of behavioral laboratory tasks that have generated considerable research interest the past centennial is the relative simple task of moving a finger or hand to an auditory sequence consisting of clicks or tones (e.g., finger tapping). It was introduced in early experimental psychology studies [1], [2], and the defining feature is repetitive movements alternating back and forth from a single contact point or between different contact points. These points consist of a period of negligible movements, and the goal of the task is to arrive at these points in accordance with the isochronous (repetitive) metronome signal. The task can be divided into two distinct phases: Synchronization that consists of actively moving in accordance with the metronome, or continuation where the metronome is turned off and the participant attempts to maintain a previously provided metronome rhythm for a given period [3], [4]. In the first case (synchronization phase), the task involves temporally coordinating motor responses with predictable external events. Hence, it falls under the term sensorimotor synchronization which is typically defined as the rhythmic coordination of perception and action [5], [6].

Perhaps for their apparent simplicity, synchronization tasks form one of the backbones in the study of human timing abilities. In its dictionary form, the word ‘timing’ is defined as “*the ability to determine or regulating the precise occurrence (time) of a series of actions or events to achieve a desired or optimum effect”* [7]. Given that regulation and control of serial behavioral responses might occur in many contexts, a more precise term for tasks involving explicit production of responses in accordance with a metronome is motor timing [8], [9]. The process of regulating the precision of the motor output with respect to the metronome input in motor timing tasks is considered to depend upon temporal processing, a term commonly defined as decoding of temporal information [10]. The construct includes the ability to process, segregate and detect the temporal structure of incoming stimuli [11], [12]. Motor timing tasks are considered to be some of the possible behavioral assessments of this processing dimension, and responses obtained from motor timing tasks can be utilized as behavioral indices of temporal processing abilities [13], [14].

Performance in motor timing tasks where responses are synchronized to a regular metronome is considered to be the result of primary ‘automatic’ processing in which temporal variability is inaccessible to conscious manipulation [5]. In particular, it is hypothesized that motor production of temporal intervals <1 sec (that appears repetitively in continuous succession) favors an automatic rather than a cognitive mode of temporal processing [15]. This is based upon a consistent picture from meta-analysis of the neuroimaging literature, in which motor timing in the range of milliseconds loads little on brain regions known to be involved in cognitive control [16]. Behavioral studies have further demonstrated little conscious control of motor timing performance: perturbations in timing performance by distracting sounds occur involuntarily and unconsciously [17], [18], and subliminal perturbations in the pacing stimuli are tracked without participant’s awareness [19]–[22]. Furthermore, dual-task paradigms have shown that timing performance is marginally affected by simultaneously performing other cognitive or motor tasks [23]. A logical entailment of these findings is that performance in motor timing tasks, as an index of temporal processing ability, is not strongly mediated by individual differences in other executive functions such as attention to tasks or manipulation of information in working memory [24], [25].

In studies applying information-processing models and methods, measures of intelligence have been found to be correlated with measures of mental speed obtained from elementary cognitive tasks [26]. These mental speed studies resemble the major experimental approach towards elucidating the basic cognitive mechanisms underlying individual differences in psychometric intelligence (or *g*) [27], [28]. Within this conceptual framework, information-processing speed (IPS) is proposed as a basic parameter of cognitive functions [29]. In addition to the consistent relationship between psychophysical tasks capturing processing speed and more complex cognitive tasks [30], [31], psychometric measures of IPS correlate with performance in a broad range of cognitive domains [32]. On the whole, all these accounts are typically interpreted within a general framework that considers fast, accurate and efficient processing of information a fundamental characteristic of the central nervous system and a basic determinant of individual differences in cognitive abilities [33], [34]. Under this perspective, psychophysical and psychometric measures of information processing speed are considered as valid indicators of neural efficiency [35].

It is still an open question as to what constitutes the biological basis of mental speed-intelligence relations. Temporal processing, in the form captured by motor timing tasks, has also long been considered as a mechanism associated with the brains basic neural design features [36]–[38]. It is hypothesized to capture a fundamental physiological process associated with temporal neural resolution, linked to hypothetical oscillatory processes in the brain [39], [40]. This viewpoint is related to the concept of a metaphorical internal “clock” mechanism in the central nervous system associated with coordination of different neural activities, originally proposed by Surwillo [41]. Motor timing tasks, and other psychophysical timing tasks assessing timing accuracy, have been advanced as putative behavioral measures of temporal resolution in the central nervous system due to independency of factors such as motor response times [42], [43] and, mentioned above, non-temporal cognitive operations [39], [44].

One approach towards explaining processing speed-cognitive ability relations is therefore to consider temporal stability or resolution of neural activity as biological substrate involved in basic cognitive processing, as well as influencing the speed and efficiency of information processing [40], [45]. Under this perspective, it is assumed that more efficient temporal processing is associated with an ability to perform and coordinate a specific sequence of mental operations faster. This in turn, ultimately predicts higher levels of performance on complex cognitive tasks. The relationship between performance in cognitive tasks and processing speed measures is therefore hypothesized to result from quantitative individual differences in timing abilities that can be captured by motor timing tasks.

Indeed, Madison, Ullen and co-workers have demonstrated that performance variability in a simple motor timing task, what that they term “isochronous serial interval production”, is correlated (mean *r* = .3) to non-verbal ability measures (Raven's Progressive Matrices and the Wiener Matrizen Test) and measures of simple/two-choice reaction time performance [24], [25], [45], [46]. The research originating from this group has provided evidence for timing or temporal processing as a mediating factor in the relationship between cognitive abilities and mental speed: motor timing variability explained both shared cognitive-speed variance as well as unique variance of the non-verbal ability measures [24], [46].

In this study, we further investigated the hypothetical role of motor timing abilities in explaining IPS-cognitive ability relations. To this end, a sample of university students (*n* = 100) performed motor timing tasks and psychometric measures including information processing speed and Raven’s Progressive Matrices. An important aspect of this study was that we also included a test battery for fine motor skills. This allowed for assessing several sub-components of the theoretical accounts concerning timing abilities outlined above. First, timing is an inherently critical aspect of movement control involving activating motor units at the correct times on the order of tens of milliseconds and represents a clear example of an inherently timing-intensive computation in this timescale [11], [47]. Based upon this simple observation, one might expect significant relations between motor timing performance and motor skill when both are obtained with the same effector and require temporal and spatial accuracy. However, there is little evidence pertaining to this hypothesis. Indirect evidence for concurrent timing deficits and poor motor skills have been found in pediatric sub-populations, but the overall heterogeneity in motor performance of these children accompanied by conflicting findings makes it impossible to draw any firm conclusions [48], [49]. Secondly, motor tasks performed under a time limit also require (amongst other things) fast information-processing [48]. Motor skill tests can therefore incorporate a mental speed component that is hypothetically not captured by motor timing tests. Based upon these considerations one might propose an alternative hypothesis: IPS and motor timing abilities are both correlated to psychometric intelligence, but only psychometric IPS explains any variance in fine motor skills.

## Methods

### Ethics Statement

The experimental procedures were initiated following approval of the protocol by the central regional ethics committee for medical research (REC Central). All subjects provided written consent prior to participating in the study and all procedures were carried out in accordance with the code of Ethics of the World Medical Association (Declaration of Helsinki).

### Participants

Participants were recruited from the university college community. 37 men and 63 women with a mean age of 22.6 years (SD 2.6) across the entire sample participated in the study. All were neurologically healthy and reported very little musical experience (none were professional musicians). Using Oldfield’s procedure [50], 93% of participants were defined as right-hand dominant.

### Psychometric Tasks

The standard paper-and-pencil version of the *Raven’s Standard Progressive Matrices* (R-SPM) was used to assess non-verbal ability [51]. This test mainly measures psychometric general intelligence [27]. Participants were given a booklet with 60 two-dimensional pattern-matching matrices in which one small section is missing, and asked to choose amongst six options the one they perceived fit for the missing section. The items become progressively more difficult. There was no time limit for completing the booklet, and the raw score for number of correct matrices was used for further analysis.

Processing speed was assessed with the *Digit Symbol Coding* and *Symbol Search* subtests of the Wechsler Adult Intelligence Scale-III [52]. In the digit symbol coding task, participants were presented with a key that associated the digits one to nine with distinct symbols. They were asked to go through a list of digits arranged in rows on a paper sheet, and copy the corresponding symbol underneath each digit with a pencil. The number of digit–symbol pairs that were correctly completed under a two-minute time limit served as the index of performance. In the symbol search subtask, the participant indicated with a pencil whether one of two target geometric symbols on the left of a row also appears among a row of five geometric symbols printed to the right. The number of correctly identified symbols under a two-minute limit served as measure of performance. A sum-score of these two tasks was used as an index of information-processing speed.

### Fine Motor Skills

The standardized *Purdue Pegboard Battery* was used as an assessment of fine motor skills and administered according to the instructions in the test manual [53]. The pegboard (model 32020, Lafayette instrument, US) had two rows of 30 holes. Participants were asked to take pegs of 1 mm diameter and 25 mm in length from a bowl at the top of the pegboard and place them in the row of holes indicated by the tester. Following a series of practice trials, participants were given 30 s to place as many pegs as possible; first with their dominant hand, then with their non-dominant hand and finally with both hands together. The score reported was the number of pegs placed for each respective condition. In the assembly subtask, participants were asked to put together an assembly of a peg, a collar and two washers, working with both hands together. In this subtask participants were given one-minute to complete as many “assemblies” as possible. The score reported was the number of parts assembled. A sum-score of the four pegboard subtasks was calculated to obtain a composite measure of fine motor skill.

### Motor Timing Task

Complete details of the timing task can be found elsewhere [54]. Each participant was tested individually, sitting comfortably at a small table (height: 60 cm×width: 100 cm×depth: 70 cm). In each trial, the participant synchronized dominant-hand tapping movements to an isochronous auditory metronome (Fine Metronome 3.4 software, Fine Software Inc., USA) comprised of clicks presented through two loudspeakers (Inspire T10, Creative Labs Inc., USA), each positioned in a corner of the table. During measurements the metronome was on for a period of 30 s, partitioned into 10 s of adaptation and 20 s of sampling. The subjects were not aware of the transition point between the adaptation and sampling phases. Responses were given by hitting three 10×7 cm Pad switches (Pal Pad, Inclusive Technology Ltd, UK) spaced 20 cm apart to form an equilateral triangle. Two switches were positioned directly in front of the subject, 10 cm from the near edge and 35 cm from each side of the table. The switches responded to ∼ 0.34 Newton’s of force and created only minimal sound upon impact. Participants were explicitly instructed to follow the metronome when it was activated; by hitting the pads with the index finger one-by-one moving towards the body midline (medial direction) so their movements resembled the shape of a triangle. Prior to synchronization trials, the task was demonstrated and participants practiced for 30 s without the presence of any external pacing stimuli. Movements were synchronized to four different metronome inter-stimulus intervals (500, 650, 800 and 950 ms) with the order of stimulus presentation fully randomized.

Raw analogue data containing behavioral responses and metronome pulses were obtained with the Qualisys Track Manager software and exported to Matlab 7.8 (Mathworks, USA) for further processing. Signals from individual trials were filtered by removing values smaller than two standard deviations from the mean. As typical for motor timing experiments in which movements are synchronized to metronome beats, we operationalized performance by measuring the precision of the response-pacing of individual trials. These so-called synchronization errors between responses and stimulus were computed by subtracting the measured onset time of each auditory stimulus from the registered time of the nearest response, so that a negative synchrony signified that the response preceded the stimuli. In each individual trial the mean and variability (SD) of 15 synchronization errors were calculated. Against the background of little inter-trial variability in performance [54] and high correlations between metronome rates in the motor timing task [55], a metric used for further analysis was the mean of these measures across all trials (*n* = 4) within each subject.

### Data Analysis

The distribution of cognitive, fine motor and timing scores in the dataset was investigated with Kolmogorov-Smirnov tests, histograms and Q–Q plots. Relationships between variables were examined with Pearson product-moment correlations. Regression and commonality analysis was performed to determine the proportion of variance in Raven’s scores associated with timing and IPS scores uniquely, as well as with common effects of these variables. Similarly, commonality analysis was performed to determine the proportion of shared or unique variance in fine motor scores associated with timing and IPS scores. Statistical analysis was conducted with PASW statistics 20.0 (IBM, New York, US) and *P*<.05 was used as significance criterion.

## Results

Descriptive statistics of all study variables can be found in table 1. Raw Pearson’s product-moment correlations between timing scores, information-processing speed, Raven's and fine motor scores are depicted in table 2. These indicate significant correlations between mean synchronization error (mean *r* = .56) and between synchronization variability (mean *r* = .65) from motor timing conducted at different ISIs. The average correlation (*r* = .19) between mean and variability of synchronization errors was not significant. Significant correlations can also be found between pegboard tasks (mean *r* = .4) and between IPS tasks (r = .5), as well as between IPS measures and Raven's scores (mean *r* = .2). Mean synchronization error was significantly correlated to IPS and Raven’s scores (mean *r* = .3) but not to pegboard scores. The correlation coefficients for relations between synchronization variability and fine motor/cognitive measures were not significant.

**Table 1:** Descriptive statistics for the entire sample (*N* = 100) on cognitive, fine motor and motor timing measures.

Test	Mean (SEM)
Raven’s Progressive Matrices	52.43 (0.45)
Symbol Search	39.13 (0.63)
Digit Symbol Coding	88.25 (1.36)
IPS sumscore	127.38 (1.76)
Purdue Pegboard - Right	16.45 (0.18)
Purdue Pegboard -Left	14.77 (0.16)
Purdue Pegboard - Bilateral	24.93 (0.32)
Purdue Pegboard - Assembly	36.70 (0.59)
Purdue Pegboard - Sumscore	92.85 (0.98)
*Mean of synchronization errors (ms)*	
ISI 500	−11 (2)
ISI 650	−10 (2)
ISI 800	−10 (3)
ISI 950	−12 (3)
Mean score	−10 (2)
*Variability of synchronization errors (ms)*	
ISI 500	18 (2)
ISI 650	23 (2)
ISI 800	28 (2)
ISI 950	32 (3)
Mean score	25 (2)

**Abbreviations**

**SEM** Standard error of the mean.

**IPS** Information-processing speed.

**ISI** Inter-stimulus interval.

**Table 2:** Raw Pearson product-moment correlation coefficients between motor timing performance, fine motor scores, measures of information-processing speed and the Raven’s test.

		1	2	3	4	5	6	7	8	9	10	11	12	13	14	15
1. Motor timing (mean)	500	**1**	**.57**	**.47**	**.33**	.03	.18	.01	.07	.04	.02	.04	.08	.01	.14	.12
2.	650		**1**	.**70**	**.60**	**.36**	**.29**	**.34**	.08	.07	.01	.16	**.27**	**.25**	**.28**	**.28**
3.	800			**1**	.**67**	**.24**	**.25**	.17	**.26**	.17	.04	.07	.15	**.24**	**.22**	**.22**
4.	950				**1**	.17	**.20**	.13	.**37**	.05	.13	.10	.06	.16	**.22**	**.29**
5. Motor timing (variability)	500					**1**	**.80**	**.62**	**.61**	.01	.07	.02	.15	.19	.09	.14
6.	650						**1**	**.70**	**.62**	.06	.12	.09	.06	.17	.11	.11
7.	800							**1**	**.56**	.06	.11	.03	.15	.01	.03	.10
8.	950								**1**	.15	.09	.07	.15	.08	.04	.07
9. Pegboard	Right									**1**	**.34**	**.41**	**.35**	**.21**	.14	.06
10	Left										**1**	**.42**	**.40**	.18	.15	.02
11	Both hands											**1**	**.48**	.11	.03	.10
12	Assembly												**1**	**.23**	.15	.01
13. Digit symbol coding														**1**	**.51**	**.20**
14. Symbol search															**1**	**.20**
15. Raven’s test																**1**

Correlation coefficients in **bold** = *p*<.05.

In order to further address the potential shared variance between timing scores and information-processing speed in the observed relations to Raven's scores, we performed a commonality analysis with information-processing speed and mean synchronization error as independent variables. Both variables explained unique variance in the Raven's test, with 55% of the total variance explained arising from the mean timing error. The commonality between the mean timing error and IPS contributed little to the explained variance with only 2.7% of the proportion of the total variance. The total R^2^ for this model was 10.5% [F (1, 99) = 5.68; *P* = .005].

Further commonality analysis with fine motor score as the dependent variable, and with information-processing speed and mean timing error as the independent variables, indicated that 87% of the total explained variance could be attributed to information-processing speed. Timing score contributed to <1% of the proportion of total explained variance. The commonality between the two variables contributed to 12% of the total explained variance, which in the regression model of fine motor skill was 5.9% [F (1, 99) = 3.09; *P* = .05].

## Discussion

In this study, individual differences in behavioral measures of motor timing were investigated in relation to fine motor (Purdue pegboard test), information-processing speed (IPS) and Raven’s scores. Our results showed that (see table 2) timing performance, operationalized by the mean response-pacing error, was correlated to Raven's scores and the information-processing speed index. We did not find a significant correlation between fine motor scores and measures obtained from the motor timing tasks. Furthermore, information-processing speed (IPS) and timing measures explained 10% of the variance in Raven’s scores with more than half attributed to timing performance (see table 2). Only IPS explained any variation (5%) in fine motor scores. There was little shared variance explained in relation to motor and cognitive skill between IPS and timing scores (<3%).

### Relations between Motor Timing and Cognitive Performance

Correlation analyses revealed that mean synchronization error was correlated to Raven's score and our index of processing speed (see table 2 & 3). The average *r* in our study for the relation between a timing score and a cognitive variable was.3, which is remarkably similar to the results obtained by the Madison & Ullen group: Their weighted mean average *r* across several different timing-scores×intelligence test relations from two different samples is.3 [25]. These results converge with studies on different sets of behavioral timing measures (although they shared the requirement for precise timing) and their relation to cognitive performance. Specifically, Rammsayer and co-workers have demonstrated that performance in psychophysical tasks that involve maintenance and manipulation of temporal information (temporal processing) at the millisecond level is better correlated with general cognitive ability compared to information-processing speed. Furthermore, the research originating from this group has demonstrated that the portion of overall variability in psychometric *g* explained by the processing speed tasks almost entirely represented variance also explained by the temporal information processing tasks [39], [40], [56]–[62]. The latter finding is also observed in our study and that of the Madison & Ullen group, as motor timing performance explains unique as well as shared variance (with IPS measures) in non-verbal ability measures of intelligence. This allows for the strong assumption that performance in motor timing tests, as behavioral measures of temporal processing, provides explanatory value in terms of mental speed-cognitive ability relations.

**Table 3:** Commonality analysis of associations between mean synchronization error (MSE) and information-processing speed (IPS) on Raven’s scores or fine motor scores (FMS).

	Variance explained (*R* ^2^)		Proportion of total explained variance (%)	
	Raven’s score	FMS	Raven’s score	FMS
*Unique contributions*				
IPS	0.023	0.052	22.11	86.92
MSE	0.055	0.001	52.19	0.92
*Commonality*				
IPS, MSE	0.027	0.007	25.70	12.16
Total	0.105	0.059	100	100

In this study, we were not able to replicate previously documented findings of significant relations between measures of variability in elementary psychophysical tasks and cognitive performance. E.g., in the work by Madison & Ullen and co-workers variability measures from their isochronous serial interval production task were systematically correlated to intelligence [45]. However, it is not given that measures of dispersion capture performance in all timing tasks. In the Madison & Ullen studies, responses were obtained from continuation trials after the metronome is turned off and it might be expected that performance in such task has to be operationalized with measures of response variability. In our motor timing task, participants were explicitly instructed to synchronize their movement to the timing target. Under such task conditions, the participants try to maintain their motor responses close to the metronome rhythm and the average synchrony error might therefore be an important aspect of timing performance [4], [5]. Furthermore, measures of inter-individual variability in motor timing tasks has been shown to demonstrate considerable task-specificity [63]–[65] and it is not given whether variability affects or facilitates performance in tasks that require motor coordination [66]. Indeed, performance obtained from our fastest timing condition (inter-stimulus interval of 500 ms) was not systematically correlated to any of our cognitive measures (table 2). Although this finding might be explained by postulated shifts in underlying mechanisms and strategies when the ISIs are increased or decreased [15], [45], it clearly demonstrates that further experimental and differential research is needed to establish the patterns of correlations that fully capture the relationship between aspects of motor timing performance and assessments of cognitive skills.

### Relations between Motor Timing and Fine Motor Skills

In our data, performance in the fine motor skill battery (Purdue pegboard) was significantly related to IPS but not to motor timing scores (See table 2 & 3). In a study on speech and language impaired and matched control children, it was also found that pegboard scores and motor timing scores are not related [67]. This supports our hypothesis that although conducted with the same effector, motor timing measures are not related to performance in tasks considered to capture fine motor dexterity. Such tasks require other aspects of fast information-processing speed, however, as indicated by the 5% explained variance in fine motor skill by our IPS measure (see table 3). These findings suggest that motor timing tests and assessments of fine motor skills capture different aspects of human performance. Given that performance in our pegboard measure might also involve other executive functions such as sustained attention and spatial working memory, our null result regarding fine motor-timing relations can be interpreted as further evidence for relatively little impact of these cognitive processes in motor timing tests. However, other research involving pediatric sub-populations has found evidence for co-existing poor motor skills and timing abilities [48], [49]. These apparently contradictory results could be explained by methodological differences however, and will motivate further studies on the relation between individual differences in motor skills and behavioral measures of temporal processing in different sub-populations across the human life-span.

### Potential Neural Correlates of Motor Timing Abilities

Overall, the behavioral data from this and other studies [24], [25], [45], [46] indicates that motor timing measures of temporal processing can explain unique variance in performance across cognitive measures. Viewed in the light of a cognitive information-processing approach, this can be interpreted in lines of potential neural correlates. Given that any such discussion based solely on behavioral data requires precaution, theoretical perspectives have advanced that performance in tests that require synchronizing movements to a repetitive metronome, as adopted in this study, requires the operation of a centralized timing process (a metaphorical “neural clock”) functioning relatively independently of the precise parameters of the motor task [3], [4], [13], [38]. The robust findings of shared and unique variance between information-processing speed, motor timing abilities and different cognitive assessments (Raven's Progressive Matrices and the Wiener Matrizen Test), can therefore be attributed to basic neural features associated with the motor timing process. Behavioral measures of timing performance is therefore hypothesized to capture a neural mechanism that is jointly involved in achieving temporal accuracy in psychophysical timing tasks as well as in processes of importance for cognitive tasks [39], [40], [59].

The hypothetical biological underpinnings of timing (or temporal processing), measurable as behavior in simple motor timing tasks, have centered on the fact that simultaneous interaction and activation of widespread and multiple sub-cortical and cortical networks rely upon temporal precision or resolution of neural signals [68]. Indeed, substantial literature has demonstrated that coordination of neuronal activity in the millisecond range, within and between brain regions, is essential for a broad range of cognitive functions [69], [70]. Precise and reliable timing of neuronal firing is therefore theoretically important for cortical information processing [71], [72]. High temporal stability of neural activity, which might be reflected in high precision on motor timing tasks, can be associated with a generally increased capacity to form temporally well-coordinated activity in neural networks. Under such a view, it appears conceivable that individual differences in temporal precision of neural activity could influence both performance in cognitive tasks and performance in simple motor timing tasks. This “neural coordination” hypothesis might provide a parsimonious explanation of our findings concerning motor timing-cognitive performance relations (see table 1 & 2). Indeed, recent findings suggest that precise motor timing is associated with increased functional interaction between sub-cortical and cortical areas [73], [74]. Whether higher temporal precision or resolution in CNS activity leads to better coordination of specific mental operations and enable an individual to perform motor timing and cognitive tasks more accurate, is an important question for further research.

This study has several limitations that will motivate further work. As is the case of this study and in the work originating from the Madison-Ullen group, only single cognitive measures of intelligence (Raven's Progressive Matrices and Wiener Matrizen Test) have been applied when considering relations between psychometric intelligence and performance in motor timing tasks. Although these are highly correlated to psychometric *g* [27], an important avenue for further research is to incorporate test batteries that allow for direct calculation of this factor. Furthermore, other cognitive and motor performance domains should be studied to fully distinguish motor timing tests from other executive and processing functions. Also, a necessary next step in researching individual differences in motor timing abilities is to obtain larger samples that allow for testing theoretical models with structural equation modeling, which should include a wider range of motor timing measures (e.g., both synchronization and production tasks) compared to the relatively limited set of tasks included in this study. Furthermore, it is important to consider that although we observed significant relations between timing ability and cognitive measures, the unique explained variance from timing abilities upon the Raven's test was relatively low (see table 3). This is perhaps not surprising. The biological basis for individual differences across performance domains might involve hundreds of different components, each providing a small contribution to the variation in cognitive performance of such tests as the Raven's SPM.

### Conclusion

This study provides further evidence for significant correlations between performance in motor timing tests and cognitive tasks. Furthermore, timing performance was not associated with individual levels of fine motor skill. In total, timing performance explained 6% of the unique variance in a Raven's test. The present findings suggest that relations between different types of cognitive and motor timing tasks are in part dependent upon similar factors influencing performance across these performance domains, which are unlikely to reflect factors associated with individual differences in fine motor skills.
